# Artificial intelligence in radiology: 100 commercially available products and their scientific evidence

**DOI:** 10.1007/s00330-021-07892-z

**Published:** 2021-04-15

**Authors:** Kicky G. van Leeuwen, Steven Schalekamp, Matthieu J. C. M. Rutten, Bram van Ginneken, Maarten de Rooij

**Affiliations:** 1 Department of Medical Imaging, Radboud university medical center, P.O. Box 9101, 6500 HB Nijmegen, The Netherlands; 2 Department of Radiology, Jeroen Bosch Hospital, ‘s-Hertogenbosch, The Netherlands

**Keywords:** Artificial intelligence, Radiology, Device approval, Evidence-based practice

## Abstract

**Objectives:**

Map the current landscape of commercially available artificial intelligence (AI) software for radiology and review the availability of their scientific evidence.

**Methods:**

We created an online overview of CE-marked AI software products for clinical radiology based on vendor-supplied product specifications (www.aiforradiology.com). Characteristics such as modality, subspeciality, main task, regulatory information, deployment, and pricing model were retrieved. We conducted an extensive literature search on the available scientific evidence of these products. Articles were classified according to a hierarchical model of efficacy.

**Results:**

The overview included 100 CE-marked AI products from 54 different vendors. For 64/100 products, there was no peer-reviewed evidence of its efficacy. We observed a large heterogeneity in deployment methods, pricing models, and regulatory classes. The evidence of the remaining 36/100 products comprised 237 papers that predominantly (65%) focused on diagnostic accuracy (efficacy level 2). From the 100 products, 18 had evidence that regarded level 3 or higher, validating the (potential) impact on diagnostic thinking, patient outcome, or costs. Half of the available evidence (116/237) were independent and not (co-)funded or (co-)authored by the vendor.

**Conclusions:**

Even though the commercial supply of AI software in radiology already holds 100 CE-marked products, we conclude that the sector is still in its infancy. For 64/100 products, peer-reviewed evidence on its efficacy is lacking. Only 18/100 AI products have demonstrated (potential) clinical impact.

**Key Points:**

*• Artificial intelligence in radiology is still in its infancy even though already 100 CE-marked AI products are commercially available.*

*• Only 36 out of 100 products have peer-reviewed evidence of which most studies demonstrate lower levels of efficacy.*

*• There is a wide variety in deployment strategies, pricing models, and CE marking class of AI products for radiology.*

**Supplementary Information:**

The online version contains supplementary material available at 10.1007/s00330-021-07892-z.

## Introduction

The number of artificial intelligence (AI) products for radiology has rapidly expanded over the past years. The number of AI exhibitors at the yearly meeting of the Radiological Society of North America (RSNA) and European Congress of Radiology (ECR) has almost tripled from 2017 to 2019 [1, 2]. Even though the supply of AI software for radiology is vast and many radiologists seem willing to adopt and adapt AI tools, clinical implementation remains limited [3–5].

It has been shown that the extent in which research algorithms have been validated varies widely [6–8]. Only 6% of the recent papers on medical deep learning algorithms included a validation on independent external data [6]. The available evidence for commercially available AI software has not been studied yet. We focus on commercial products specifically as these are the products that are currently available for clinical use.

With this study, we aim to increase market transparency and contribute to the safe and well-considered implementation of AI software in radiology departments. We present an overview of commercially available (CE-marked) AI products for radiology. For each of these products, we collected and reviewed the scientific evidence demonstrating the efficacy of the AI software.

## Materials and methods

### Product overview

Artificial intelligence is defined by the ISO/IEC TR 24028:2020 as the “capability of an engineered system to acquire, process and apply knowledge and skills” [9]. Within this scope, we focus on image analysis products which use techniques such as machine learning and deep learning. To establish an overview of AI software products, exhibitor lists from RSNA and ECR and marketplace offerings were reviewed. Also, news sources were monitored for the appearance of new vendors, products, or certifications. In Europe, the CE mark is a prerequisite for medical devices to be allowed on the market; therefore, CE marking of the product by April 2020 was a requirement for inclusion. Also, the product had to be vendor neutral and aid the radiologist in image interpretation in clinical practice. We excluded software used for dictation or image reconstruction at the source. Some vendors offer “suites” incorporating different software components performing different tasks, while other vendors market these components as separate products. To perform a balanced evaluation, we considered suite components as separate products. As the market is fast moving, we maintain an overview of products on www.aiforradiology.com. Discrepancies between the included products in this article and the Web resource may be caused by updates of the website, refusal of companies to appear on the website, and stricter inclusion criteria for this review.

All vendors were contacted to verify the collected information and supplement the product specifications. We retrieved information about the organ-based subspeciality, modality, and main task of the product. Also, the date to market, method of deployment, and pricing model were gathered. The CE status was verified by collecting the CE certificates or Declaration of Conformity of the vendors; a public database does not exist yet (EUDAMED is planned for 2021) [10]. Also, the American FDA (Food and Drug Administration) approval status was gathered and confirmed with the public FDA database [11]. CE and FDA status reported in this study reflect the status in September 2020. For the most recent information, visit www.aiforradiology.com.

### Evidence

Scientific evidence for the efficacy of the AI products was gathered in two ways. First, PubMed was systematically searched by vendor and product name for peer-reviewed articles published between Jan 1, 2015, and May 18, 2020. Queries are provided in the supplementary materials, Table S1. Secondly, a manual search was performed by inspecting the vendor’s websites for listings of papers and requesting vendors to provide peer-reviewed papers. No date restriction was applied for the manual search.

Included articles were original, peer-reviewed, and in English, and aimed to demonstrate the efficacy of the AI software. Papers were included when the product name (including known former names) and/or company name were mentioned, the tool was applied on in vivo human data, and efficacy of the product was reported on an independent dataset (data on which the algorithm was not trained) [12]. Letters, commentaries, reviews, study protocols, white papers, and case reports were excluded.

Papers were assessed by two of the authors who independently screened the title, abstracts, and full paper for inclusion criteria. Cases of disagreement were resolved by the reviewers in a consensus meeting.

### Hierarchical model of efficacy

We propose an adapted hierarchical model of efficacy to categorize the papers with respect to the type of validation addressed. Originally, this model was developed by Fryback and Thornbury in 1991 as a structure to assess the contribution of diagnostic imaging to patient management [13]. It comprises six levels assessing an innovation from its technical efficacy—level 1 (does it do what it is supposed to do)—up until societal efficacy—level 6 (how do the costs and benefits compare). We have adapted the definitions and split level 1 into two subtypes to better accommodate the appraisal of scientific evidence regarding the contribution of AI software to the diagnostic imaging process. The adapted definition of each level is given in Table 1.

**Table 1:** Hierarchical model of efficacy to assess the contribution of AI software to the diagnostic imaging process, adapted from Fryback and Thornbury (1991) [13]

Level	Explanation	Typical measures
Level 1t	Technical efficacy Article demonstrates the technical feasibility of the software	Reproducibility, inter-software agreement, error rate
Level 1c	Potential clinical efficacy Article demonstrates the feasibility of the software to be clinically applied	Correlation to alternative methods, potential predictive value, biomarker studies
Level 2	Diagnostic accuracy efficacy Article demonstrates the stand-alone performance of the software	Standalone sensitivity, specificity, area under the ROC curve, or Dice score
Level 3	Diagnostic thinking efficacy Article demonstrates the added value to the diagnosis	Radiologist performance with/without AI, change in radiological judgement
Level 4	Therapeutic efficacy Article demonstrates the impact of the software on the patient management decisions	Effect on treatment or follow-up examinations
Level 5	Patient outcome efficacy Article demonstrates the impact of the software on patient outcomes	Effect on quality of life, morbidity, or survival
Level 6	Societal efficacy Article demonstrates the impact of the software on society by performing an economic analysis	Effect on costs and quality-adjusted life years, incremental costs per quality-adjusted life year

*Level 1t* level 1, technical; *Level 1c* level 1, clinical

### Analysis

Products were categorized according to the aimed subspeciality, modality, main task, CE marking, FDA clearance, deployment method, and pricing model. We calculated the mean time between the founding of the company and bringing their first AI product to the market, excluding companies that were founded before 2005.

We report the available scientific evidence and the level of efficacy the papers addressed. Multiple levels could be assessed by a single paper. For each article, we reviewed the author list, funding source, and disclosures to categorize the publication as vendor independent or not. Data used in the included papers was categorized for the number of centers, countries, and acquisition machine manufacturers it originated from. We aggregated this information per product to give insights in the total number of centers, countries, and manufacturers addressed in the total evidence of that product.

## Results

### Product overview

The product search resulted in 100 AI solutions that are CE marked and commercially available in Europe. From 74 out of the 100 products, product information was provided by the vendors. The remaining products were analyzed using public information only.

**Fig. 1:**
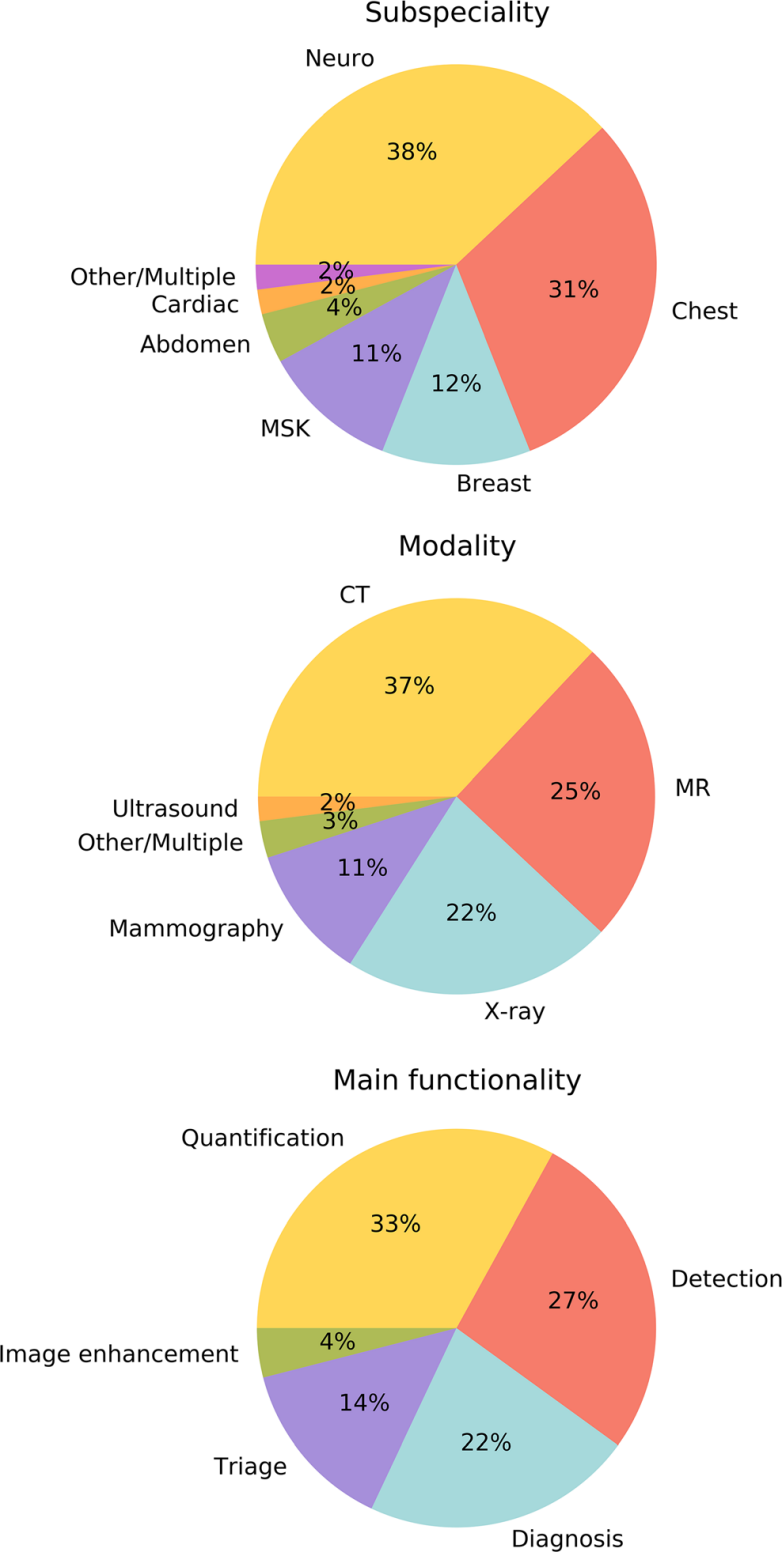
Characteristics of 100 CE-marked AI products based on organ-based subspeciality, modality, and main functionality. *MSK*, musculoskeletal

Results in Fig. 1 show that available AI software mostly address neuroradiology (38) and chest radiology (31), followed by breast (12) and musculoskeletal (11) radiology. Regarding the modalities, products are distributed over CT (37), MR (25), and X-ray (22). The main tasks performed by the software are quantification tasks (33), such as region segmentation or performing automated measurements. Out of the 53 vendors, 18 provide multiple products, of which 7 address multiple organ-based subspecialities.

**Fig. 2:**
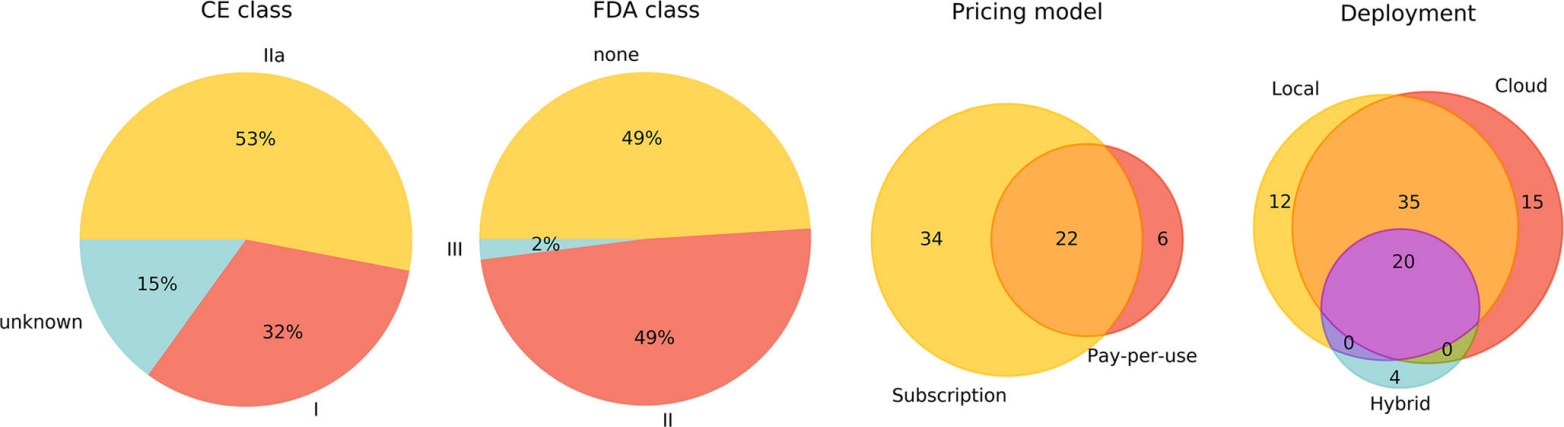
Distribution of CE class, FDA class, pricing model, and deployment strategies of 100 CE-marked AI products. *CE*, European Conformity Marking; *FDA*, Food and Drug Administration

Out of the 100 CE-marked products, 51 also have FDA clearance as can be seen in Fig. 2. QVCAD by QView Medical and ClearRead Xray - Detect by Riverain Technologies have the most stringent premarket approval (PMA), class III. All other products have class II clearance via the 510(k) pathway, except for Viz.ai which received class II clearance via De Novo premarket review pathway.

Regarding the CE mark, we observe a spread over risk class I (32%) and class IIa (53%). For the remaining 15 products, we did not receive the information from the vendor and could not find sufficient public information to determine the class.

A majority of the vendors offer their products with multiple options regarding the deployment strategy and pricing model as demonstrated in Fig. 2. Local installations (n = 67) and cloud-based (n = 70) offerings are similarly represented of which 54 products permit both. Subscription/license models are more prevalent (n = 56) than the pay-per-use pricing model (n = 28), but a large number of products (n = 22) are offered with both models.

**Fig. 3:**
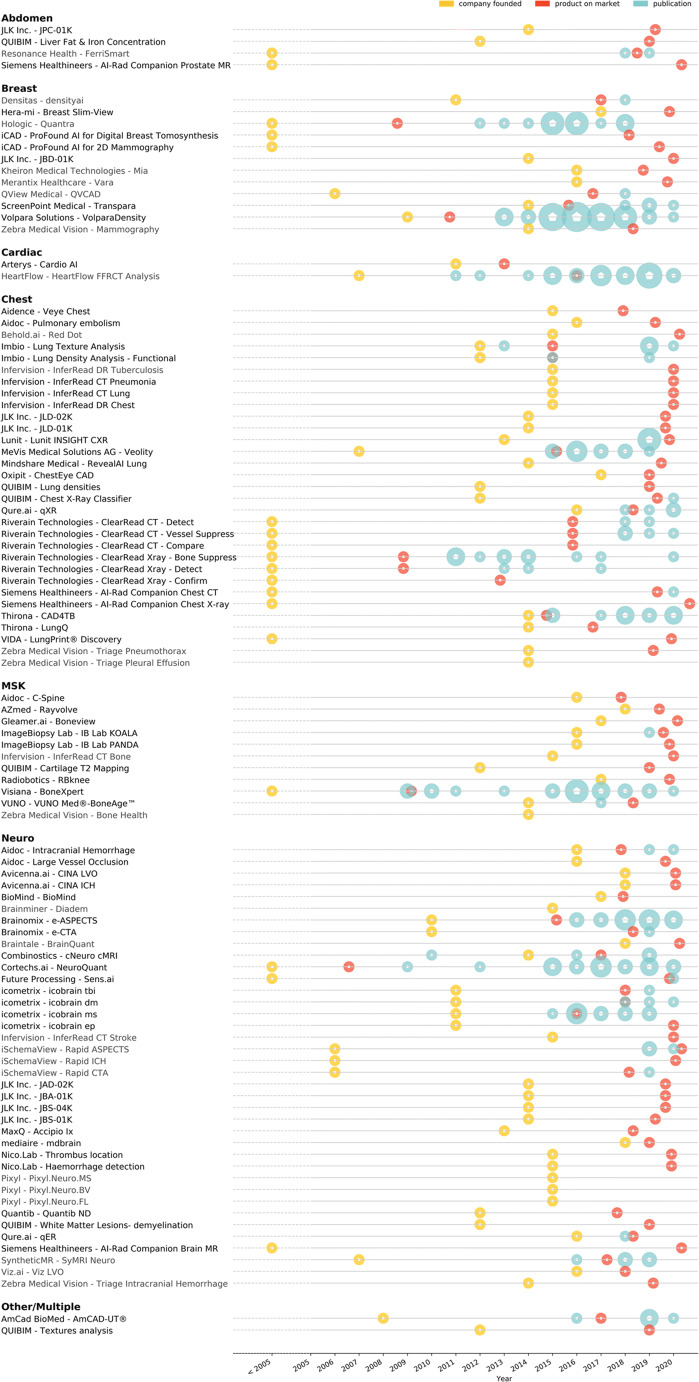
Visualization of the timeline for the one hundred CE-marked AI products. Yellow circles denote the year the company was founded, red circles the year the product was brought to market, and blue circles provide the date of peer-reviewed papers. The larger the circle, the more papers were published in that year. Product specifications were not verified by the vendor when the product is listed in gray text

From the 94 products of which we retrieved the date to market, 64 were launched between 2018 and May 2020. The mean time to market from foundation of the company to the first AI product on the market was 3 years and 11 months. The founding date of the company as well as the market release date and publications for each product are shown in Fig. 3.

For complete specifications of individual products, we refer to our public online database on www.aiforradiology.com that is regularly updated.

### Evidence

The search strategies on PubMed yielded a total of 791 papers, from which 175 met the inclusion criteria. Manual search added 62 additional papers, resulting in a total of 237 included peer-reviewed articles. The flowchart of the inclusion is displayed in Figure S1 in the supplementary materials. An article could appear multiple times in the overview when it addressed more than one AI product (n = 21). Search queries and the resulting hits are provided in Table S1 of the supplementary materials.

**Fig. 4:**
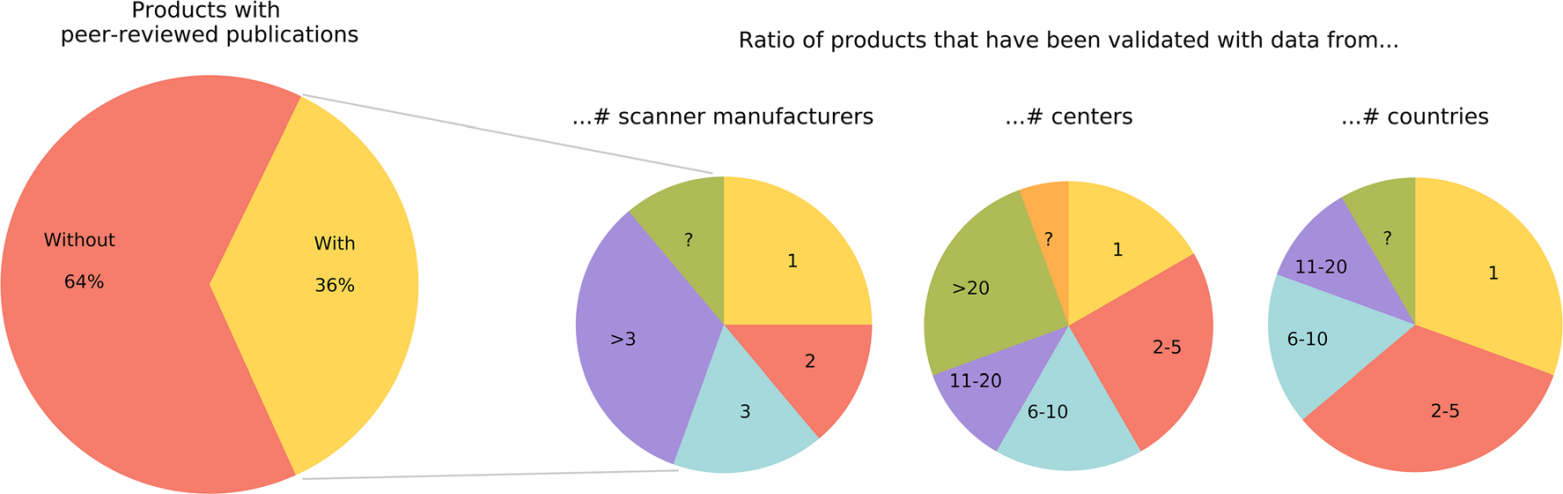
Peer-reviewed articles were present for 36 out of the 100 commercially available AI products. For these 36 products, the three pie charts on the right demonstrate the characteristics of the validation data when aggregating all included papers per product (i.e., the number of scanner manufacturers, centers, and countries)

**Fig. 5:**
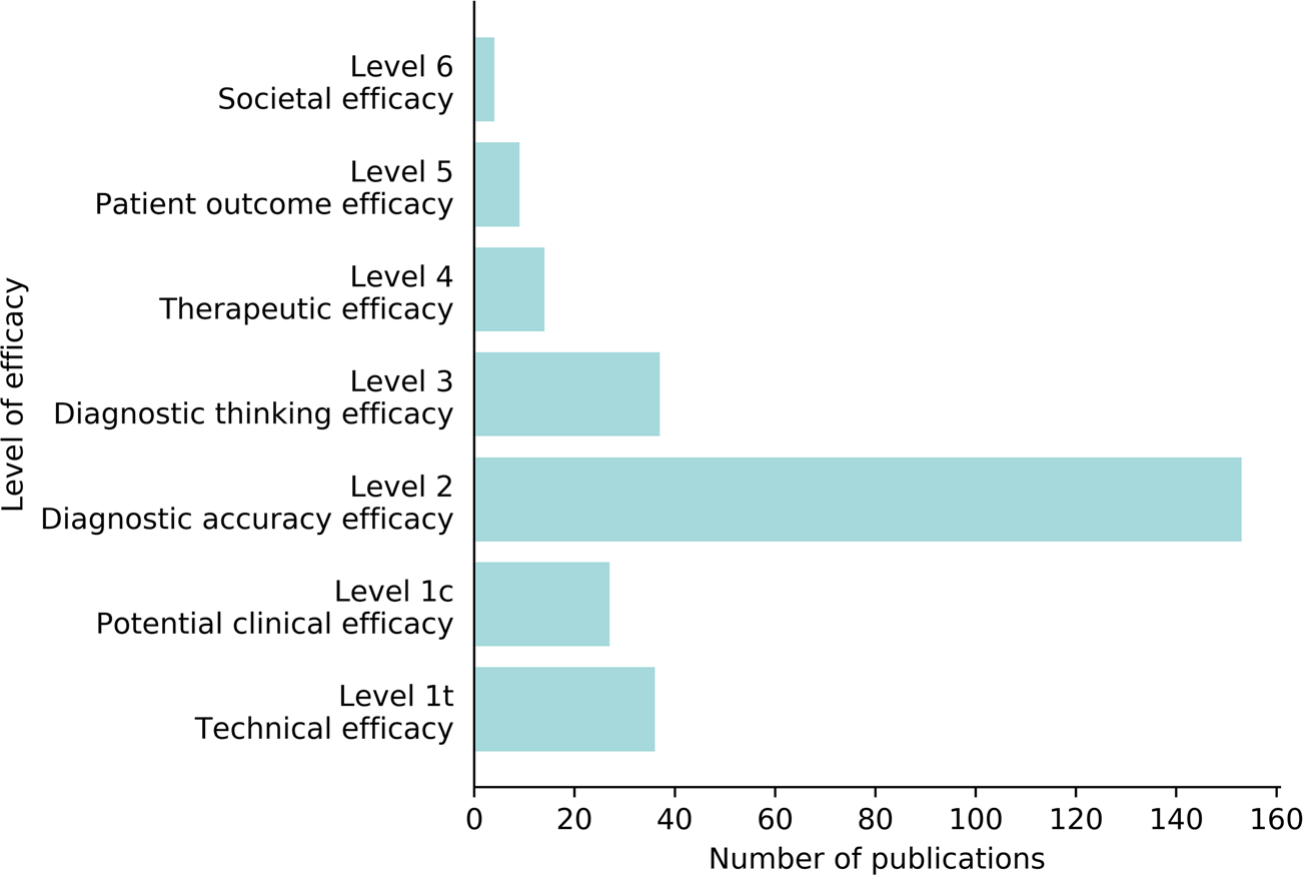
The levels of efficacy of the included papers. The search strategy yielded 239 peer-reviewed publications on the efficacy of 36 out of 100 commercially available AI products. A single paper could address multiple levels

For 64 of 100 included commercially available AI products, no scientific peer-reviewed papers via PubMed or manual search were retrieved as can be seen in Fig. 4. Figure 5 shows the evidence on the efficacy of the other 36 products. From all papers, 65% (153/237) evaluated the diagnostic accuracy efficacy (level 2), demonstrating the stand-alone performance of the product; 27% (63/237) comprised studies demonstrating either clinical or technical feasibility of the product (level 1t and 1c). Studies addressing efficacy levels 3 or higher validate aspects of the (potential) clinical impact of the AI software. From all studies, 27% (64/237), involving 18 products, evaluated the efficacy on level 3 or higher.

Almost half of the studies (116/237) were performed independently from the vendor. A vast majority of studies (192/237) were retrospective in design. Multicenter data was used in only 30% (71/237) of the studies. Data inclusion covered multiple countries in 15% (35/237) of the papers and data were acquired with scanners from multiple manufacturers in 25% (59/237) of the included studies. When aggregating all studies per product, a majority of the products with evidence on the efficacy were validated with data from multiple scanner manufacturers, multiple centers, and multiple countries as demonstrated in Fig. 4.

A summary of the article characteristics per product is provided in the supplementary materials Table S2.

## Discussion

The aim of this study was to increase transparency in the field of commercial AI software in radiology and its scientific evidence. Our results demonstrate that despite the vast amount of offerings, the market is still in its infancy. Only 36 out of the 100 evaluated CE-marked products had peer-reviewed evidence on the efficacy of the AI software. If evidence was present, the focus was mostly on technical and clinical feasibility (27%) and stand-alone performance (65%) and few studies (also) addressed the clinical impact (27%) of the software. Deployment and pricing strategies have not yet converged to a preferred standard and most vendors offer multiple options. The fact that two-thirds of the products were brought to market in the past 2 years (January 2018–April 2020) may be an explanation for this. Even though the 100 products evaluated in this study are CE marked and commercially available in Europe, for most products we await to discover the impact they have in clinical practice.

### Supply

A majority of solutions evaluated here offer an AI product to perform a specific task. In the area of stroke and oncology, we observe more “suites” aiming to address the whole diagnostic path. Only 7 vendors address multiple organ-based subspecialities with their products. For radiologists and their departments, this means that they have to deal with multiple vendors to supply their AI need for the different tasks and specialties. This implies an overhead of sales, contracts, technical integration procedures, training, and evaluation. The rise of marketplaces offered by scanner manufacturers, PACS companies, and new parties that aim to form the middleman may mitigate this problem when AI in radiology becomes more widely used.

### Regulatory approval

Some AI product overviews have previously been published [14, 15]. However, we are the first to specifically consider AI software as a medical device that is on the European market today (CE marked). We believe this is the most relevant group of products to study for two reasons. First, these are products that are available for clinical use and should have passed the initial research and development phase. Second, the regulatory approval system of medical devices in Europe is not transparent. There is no public database available to verify certification or the clinical validation on which the certification was based, such as the American counterpart, FDA, does offer [10, 11]. In addition, vendors often do not clearly state on their website if their product has a CE mark, and if so which class. Through requesting this information, we aim to increase transparency of the market.

Our results revealed that almost all products are either certified as class I or class IIa under the Medical Device Directory. The classes are related to the risk of the product: class I being a low risk and class III a very high-risk medical device. Noteworthy is that similar products may be certified under different classes. We observe this, for example, among products aimed at large vessel occlusion detection, chest X-ray abnormality detection, or brain region quantification. Whereas a class I CE mark is obtained through self-certification, class II requires an external audit by a notified body, which is a more elaborate process that includes the assessment of, among other things, the validation results. The Medical Device Regulation that will replace the current Medical Device Directory as of May 2021 may change this discrepancy over classes and necessary validation as it is expected that most AI software products in medical imaging will become IIa or even IIb. It remains to be seen if this will also affect the amount of peer-reviewed evidence on the AI software.

### Evidence

In this study, we reported the available peer-reviewed evidence categorized by level of efficacy (Table 1). We proposed a hierarchical model of efficacy and this may raise the question what level is needed to prove efficacy of the product. We believe there is no general answer to this question: it is dependent on the product and its intended use. Demonstrating higher levels of efficacy may be necessary when, e.g., opting for health insurance reimbursement. However, in case the algorithm performs a measurement task that a radiologist would otherwise do, demonstrating accuracy (level 2) may be sufficient and higher level validation may merely lead to high costs for clinical studies, thus driving up the price of the software.

### Limitations

We are aware that the definition of AI or what is part of clinical radiological practice is not trivial, and therefore our product inclusion criteria are somewhat debatable. For example, products for analysis of cardiac ultrasound were excluded from this study as this is often (but not always) part of the cardiology department. We believe this study provides a general overview of what the current state of AI in radiology entails.

Unfortunately, some vendors did not respond to our request to provide information or decided to withhold some information. Therefore, for some product information on the CE class, distribution model and pricing strategy is incomplete. We completed the missing information with public data where possible.

### Future perspective

The development of EUDAMED may be an important step forward to more transparency in the medical device industry. We demonstrated that publicly available peer-reviewed evidence on the efficacy of AI products is often lacking. Preferably, the data and validations by which the AI products are CE marked would be included in the public database.

The finding that most products were brought to market in the past 2 years demonstrates that this is a dynamic and evolving market. This study provides a snapshot of the AI landscape in clinical radiology. To provide a continuous up-to-date overview of AI in radiology products, we created and maintain the website www.aiforradiology.com. It will become clear in the next few years how the AI for radiology market evolves, grows, and matures. Future research may thus entail an updated analysis to discover what and how the landscape has changed.

## Supplementary Information

ESM 1 (RAR 237 kb)

ESM 2 (PDF 235 kb)

ESM 3 (XLSX 66 kb)

## Abbreviations

AI: Artificial intelligence
CE mark: European Conformity Marking
ECR: European Congress of Radiology
FDA: Food and Drug Administration
MSK: Musculoskeletal
PMA: Premarket Approval
RSNA: Radiological Society of North America

## Declarations

### Guarantor

The scientific guarantor of this publication is Bram van Ginneken.

### Statistics and biometry

No complex statistical methods were necessary for this paper.

### Methodology

• retrospective

• observational

• performed at one institution
