# Supplementary material for: Artificial intelligence in radiology: 100 commercially available products and their scientific evidence
Source: Eur Radiol. 2021 Apr 15;31(6):3797–804. doi: 10.1007/s00330-021-07892-z (PMC8128724; doi:10.1007/s00330-021-07892-z)
Supplement: Supplementary file 2 — (PDF 235 kb) [file 330_2021_7892_MOESM2_ESM.pdf]

Artificial intelligence in Radiology; 100 commercially available products and their scientific evidence

Supplementary material

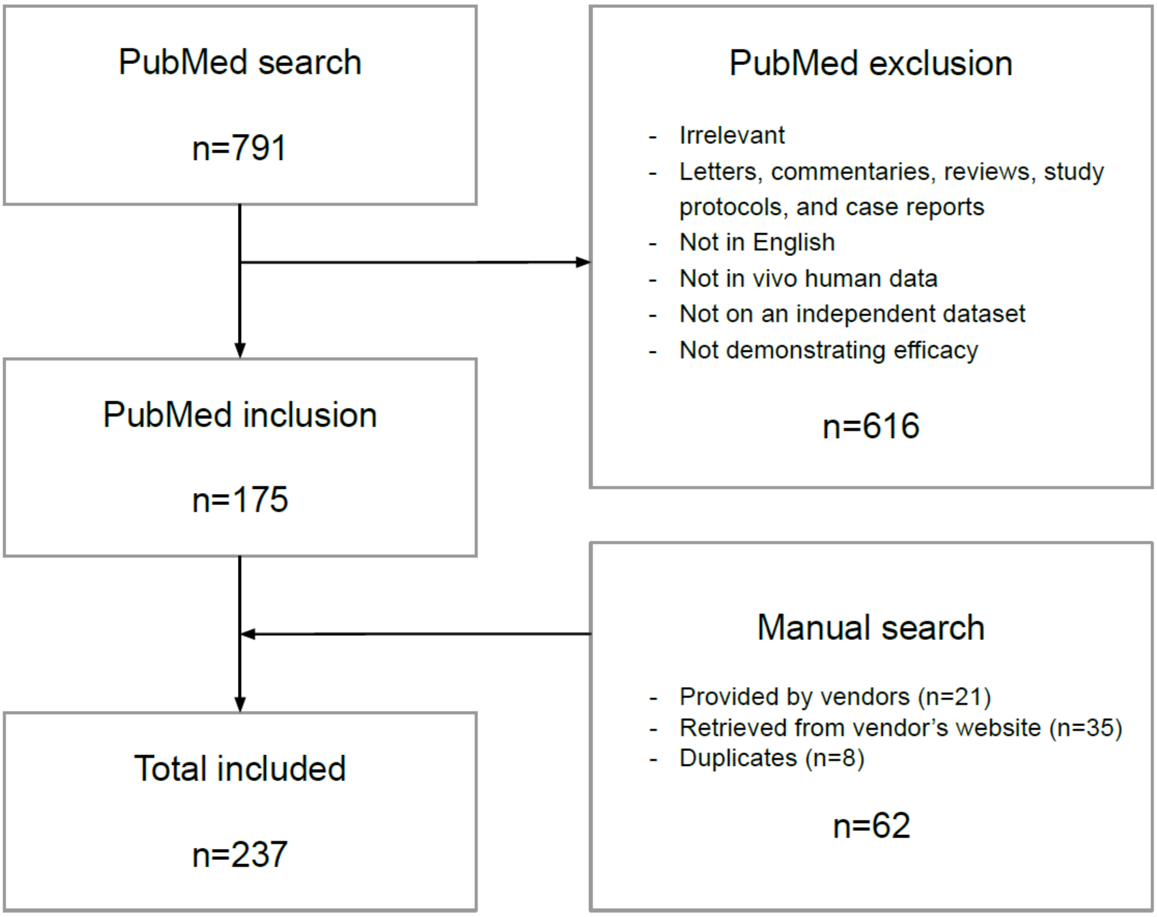

Figure S1: Flowchart of literature inclusion

Table S1: Literature search hits and inclusion per vendor

Company	Query PubMed	Hits PubMed	Included PubMed	Included manual	Included total
Aidence	(("2015"[Date - Publication] : "3000"[Date - Publication])) AND ("Aidence" OR "Veye Chest")	5	0	0	0
Aidoc	(("2015"[Date - Publication] : "3000"[Date - Publication])) AND "Aidoc"	1	1	1	2
AmCad BioMed	(("2015"[Date - Publication] : "3000"[Date - Publication])) AND ("AmCAD Biomed" OR "AmCAD-UT")	5	4	1	5
Arterys	(("2015"[Date - Publication] : "3000"[Date - Publication])) AND "Arterys"	6	0	0	0
Avicenna.ai	(("2015"[Date - Publication] : "3000"[Date - Publication])) AND (avicenna AND (LVO OR ICH) OR "CINA LVO" OR "CINA ICH")	1	0	0	0
AZmed	(("2015"[Date - Publication] : "3000"[Date - Publication])) AND ("Azmed" OR "Rayvolve")	0	0	0	0
Behold.ai	(("2015"[Date - Publication] : "3000"[Date - Publication])) AND (Behold.ai OR "Red Dot")	43	0	0	0
BioMind	(("2015"[Date - Publication] : "3000"[Date - Publication])) AND Biomind	0	0	0	0
Brainminer	(("2015"[Date - Publication] : "3000"[Date - Publication])) AND (Brainminer OR Diadem)	23	0	0	0
Brainomix	(("2015"[Date - Publication] : "3000"[Date - Publication])) AND ("Brainomix" OR "e-Stroke Suite" OR "e-ASPECTS" OR "e-CTA")	51	15	1	16
Braintale	(("2015"[Date - Publication] : "3000"[Date - Publication])) AND (braintale OR brainquant)	0	0	0	0

Combinostics	(("2015"[Date - Publication] : "3000"[Date - Publication])) AND (Combinostics OR "cNeuro cDSI" OR "cNEURO cMRI" OR "PredictND")	35	3	1	4
Cortechs.ai*	(("2015"[Date - Publication] : "3000"[Date - Publication])) AND ("Cortechs Labs" OR "NeuroQuant")	35	16	2	18
Densitas	(("2015"[Date - Publication] : "3000"[Date - Publication])) AND (Densitas OR densitasai)	1	1	0	1
Future processing	(("2015"[Date - Publication] : "3000"[Date - Publication])) AND ("Future Processing" OR "Sens.ai")	19	1	0	1
Gleamer.ai	(("2015"[Date - Publication] : "3000"[Date - Publication])) AND (BoneView OR Gleamer)	0	0	0	0
HeartFlow	(("2015"[Date - Publication] : "3000"[Date - Publication])) AND (Heartflow AND FFRCT)	37	18	5	23
Hera-mi	(("2015"[Date - Publication] : "3000"[Date - Publication])) AND ("Hera-Mi" OR "Breast-SlimView")	0	0	0	0
Hologic	(("2015"[Date - Publication] : "3000"[Date - Publication])) AND Quantra AND "breast"	20	8	9	17
iCAD	(("2015"[Date - Publication] : "3000"[Date - Publication])) AND (iCAD AND (profound OR PowerLook))	1	0	0	0
icometrix	(("2015"[Date - Publication] : "3000"[Date - Publication])) AND (icometrix OR icobrain OR msmetrix)	63	15	0	15
ImageBiopsy Lab	(("2015"[Date - Publication] : "3000"[Date - Publication])) AND ("Image Biopsy Lab" OR "IB Lab KOALA")	1	0	1	1
Imbio	(("2015"[Date - Publication] : "3000"[Date - Publication])) AND Imbio AND Lung	25	6	1	7
Infervision	(("2015"[Date - Publication] : "3000"[Date - Publication])) AND (infervision OR inferread)	13	0	0	0

iSchemaView	(("2015"[Date - Publication] : "3000"[Date - Publication])) AND (iSchemaView OR "RAPID ASPECTS" OR "RAPID ICH" OR "RAPID CTA")	40	3	1	4
JLK Inc.	(("2015"[Date - Publication] : "3000"[Date - Publication])) AND "JLK Inspection"	1	0	0	0
Kheiron Medical Technologies	(("2015"[Date - Publication] : "3000"[Date - Publication])) AND "Kheiron Medical Technologies"	4	0	0	0
Lunit	(("2015"[Date - Publication] : "3000"[Date - Publication])) AND (Lunit OR "Insight CXR")	17	5	0	5
MaxQ	(("2015"[Date - Publication] : "3000"[Date - Publication])) AND (MaxQ OR Accipio)	12	0	0	0
mediaire	(("2015"[Date - Publication] : "3000"[Date - Publication])) AND (mediaire OR mdbrain)	0	0	0	0
Merantix Healthcare	(("2015"[Date - Publication] : "3000"[Date - Publication])) AND (Vara OR Merantix) AND breast	1	0	0	0
MeVis Medical Solutions AG	(("2015"[Date - Publication] : "3000"[Date - Publication])) AND ("Fraunhofer MeVis" OR Veolity) AND (chest OR lung)	29	0	11	11
Mindshare Medical	(("2015"[Date - Publication] : "3000"[Date - Publication])) AND ("Mindshare Medical" OR "RevealAI")	1	0	0	0
Nico.Lab	(("2015"[Date - Publication] : "3000"[Date - Publication])) AND (Nico-Lab OR Nico.lab OR StrokeViewer)	6	0	0	0
Oxipit	(("2015"[Date - Publication] : "3000"[Date - Publication])) AND (Oxipit OR ChestEye)	1	0	0	0
Pixyl	(("2015"[Date - Publication] : "3000"[Date - Publication])) AND (Pixyl)	2	0	0	0
Quantib	(("2015"[Date - Publication] : "3000"[Date - Publication])) AND Quantib	10	0	0	0

QUIBIM	(("2015"[Date - Publication] : "3000"[Date - Publication])) AND Quibim	18	1	0	1
Qure.ai	(("2015"[Date - Publication] : "3000"[Date - Publication])) AND (Qure.ai OR qXR OR qER)	12	5	0	5
QView Medical	(("2015"[Date - Publication] : "3000"[Date - Publication])) AND ("Qview Medical" OR QVCAD)	3	1	0	1
Radiobotics	(("2015"[Date - Publication] : "3000"[Date - Publication])) AND (radiobotics OR rbknee)	0	0	0	0
Resonance Health	(("2015"[Date - Publication] : "3000"[Date - Publication])) AND ("Resonance Health" OR FerriSmart OR "HepaFat-Scan")	8	2	0	2
Riverain Technologies	(("2015"[Date - Publication] : "3000"[Date - Publication])) AND (Riverain OR ClearRead)	10	8	12	20
ScreenPoint Medical	(("2015"[Date - Publication] : "3000"[Date - Publication])) AND (ScreenPoint OR Transpara)	13	3	1	4
Siemens Healthineers	(("2015"[Date - Publication] : "3000"[Date - Publication])) AND ("AI-Rad Companion" OR "AI Rad Companion")	1	1	0	1
SyntheticMR	(("2015"[Date - Publication] : "3000"[Date - Publication])) AND (SyntheticMR OR SyMRI)	29	5	0	5
Thirona	(("2015"[Date - Publication] : "3000"[Date - Publication])) AND (Thirona OR CAD4TB OR LungQ)	26	10	1	11
VIDA	(("2015"[Date - Publication] : "3000"[Date - Publication])) AND LungPrint	0	0	0	0
Visiana	(("2015"[Date - Publication] : "3000"[Date - Publication])) AND (Visiana OR BoneXpert)	31	14	7	21
Viz.ai	(("2015"[Date - Publication] : "3000"[Date - Publication])) AND ("Viz.ai" OR "Viz LVO" OR "Viz CTP")	16	0	0	0
Volpara Solutions	(("2015"[Date - Publication] : "3000"[Date - Publication])) AND (Volpara OR VolparaDensity)	90	28	7	35

VUNO	(("2015"[Date - Publication] : "3000"[Date - Publication])) AND (Vuno OR BoneAge)	21	1	0	1
Zebra Medical Vision	(("2015"[Date - Publication] : "3000"[Date - Publication])) AND ("Zebra medical vision")	4	0	0	0

*Cortechs Labs name was changed to Cortechs.ai in the time between the analysis and publication

Table S2: Summary of peer-reviewed publications per product.

				**Level of efficacy**							**Data origin**		
**Vendor**	**Product**	**Papers**	**Independent**	**1c**	**1t**	**2**	**3**	**4**	**5**	**6**	**Scanners**	**Centers**	**Countries**
**Abdomen**													
Resonance Health	FerriSmart	2	2	0	2	0	0	0	0	0	2	2-5	1
**Breast**													
Densitas	densitasai	1	1	0	0	1	0	0	0	0	1	1	1
Hologic	Quantra	17	13	1	3	14	0	0	0	0	2	>20	5-10
QView Medical	QVCAD	1	1	0	0	0	1	0	0	0	1	1	1
ScreenPoint Medical	Transpara	4	0	0	0	3	1	0	0	0	>3	11-20	5-10
Volpara Solutions	VolparaDensity	35	21	5	8	20	4	2	0	0	>3	>20	11-20
**Cardio**													
HeartFlow	HeartFlow FFRCT Analysis	23	1	3	1	8	1	6	9	1	>3	>20	11-20
**Chest**													
Imbio	Lung Texture Analysis	5	2	3	0	2	0	0	0	0	2	6-10	2-5

Imbio	Lung Density Analysis - Functional	2	0	2	0	0	0	0	0	0	1	1	1
Lunit	Lunit INSIGHT CXR	5	1	0	0	5	3	0	0	0	3	11-20	6-10
MeVis Medical Solutions AG	Veolity	11	5	1	5	7	3	0	0	0	>3	>20	2-5
QUIBIM	Chest X-Ray Classifier	1	0	0	0	1	0	0	0	0	?	1	1
Qure.ai	qXR	4	3	0	0	4	0	0	0	0	2	2-5	2-5
Riverain Technologies	ClearRead CT - Detect	2	1	0	0	2	1	0	0	0	1	2-5	2-5
Riverain Technologies	ClearRead CT - Vessel Suppress	4	3	0	0	4	2	0	0	0	1	2-5	2-5
Riverain Technologies	ClearRead Xray - Bone Suppress	11	3	0	0	3	9	1	0	0	>3	11-20	2-5
Riverain Technologies	ClearRead Xray - Detect	3	2	0	0	2	2	0	0	0	>3	6-10	2-5
Siemens Healthineers	AI-Rad Companion Chest CT	1	0	0	0	1	0	0	0	0	1	?	?
Thirona	CAD4TB	11	7	0	0	11	2	1	0	3	>3	>20	6-10
**MSK**													
ImageBiopsy Lab	IB Lab KOALA	1	0	0	0	1	1	0	0	0	?	2-5	1
Visiana	BoneXpert	21	13	0	2	20	0	0	0	0	>3	>20	6-10
VUNO	VUNO Med®-BoneAge™	1	0	0	0	1	1	0	0	0	1	1	1
**Neuro**													

Aidoc	Intracranial Hemorrhage	2	2	0	0	2	0	0	0	0	2	5-10	1
Brainomix	e-ASPECTS	15	8	5	2	11	0	1	0	0	>3	>20	11-20
Brainomix	e-CTA	1	0	0	0	1	1	0	0	0	>3	2-5	?
Combinostics	cNeuro cMRI	4	1	0	0	3	0	0	0	0	3	6-10	2-5
Cortechs.ai	NeuroQuant	18	15	1	5	10	2	1	0	0	3	11-20	2-5
Future Processing	Sens.ai	1	0	0	0	0	1	0	0	0	?	?	?
icometrix	icobrain tbi	1	0	0	0	1	0	0	0	0	>3	>20	11-20
icometrix	icobrain dm	3	0	2	0	1	0	0	0	0	>3	5-10	2-5
icometrix	icobrain ms	11	3	3	4	4	0	0	0	0	3	>20	6-10
iSchemaView	Rapid ASPECTS	3	2	0	0	3	0	0	0	0	1	2-5	1
iSchemaView	Rapid CTA	1	0	0	0	1	0	0	0	0	1	1	1
Qure.ai	qER	1	0	0	0	1	0	0	0	0	0	6-10	1
SyntheticMR	SyMRI Neuro	5	3	1	4	0	0	0	0	0	3	2-5	2-5
**Other**													
AmCad BioMed	AmCAD-UT®	5	3	0	0	5	2	2	0	0	3	2-5	2-5

The following companies were evaluated, but resulted in no included papers: Veye Chest (Aidence), Pulmonary embolism (Aidoc), C-Spine (Aidoc), Large Vessel Occlusion (Aidoc), Cardio AI (Arterys), CINA LVO (Avicenna.ai), CINA ICH (Avicenna.ai), Rayvolve (AZmed), Red Dot (Behold.ai), BioMind (BioMind), Diadem (Brainminer), BrainQuant (Braintale), Boneview (Gleamer.ai), Breast Slim-View (Hera-mi), ProFound AI for Digital Breast Tomosynthesis (iCAD), ProFound AI for 2D Mammography (iCAD), icobrain ep (icometrix), IB Lab PANDA (ImageBiopsy Lab), InferRead DR Tuberculosis (Infervision), InferRead CT Stroke (Infervision), InferRead CT Bone (Infervision), InferRead CT Pneumonia (Infervision), InferRead CT Lung (Infervision), InferRead DR Chest (Infervision), Rapid ICH (iSchemaView), JAD-02K (JLK Inc.), JBA-01K (JLK Inc.), JBS-04K (JLK Inc.), JLD-02K (JLK Inc.), JBS-01K

(JLK Inc.), JPC-01K (JLK Inc.), JLD-01K (JLK Inc.), JBD-01K (JLK Inc.), Mia (Kheiron Medical Technologies), Accipio Ix (MaxQ), mdbrain (mediaire), Vara (Merantix Healthcare), RevealAI Lung (Mindshare Medical), Thrombus location (Nico.Lab), Haemorrhage detection (Nico.Lab), ChestEye CAD (Oxipit), Pixyl.Neuro.MS (Pixyl), Pixyl.Neuro.BV (Pixyl), Pixyl.Neuro.FL (Pixyl), Quantib ND (Quantib), Liver Fat & Iron Concentration (QUIBIM), Lung densities (QUIBIM), White Matter Lesions- demyelination (QUIBIM), Textures analysis (QUIBIM), Cartilage T2 Mapping (QUIBIM), RBknee (Radiobotics), ClearRead CT - Compare (Riverain Technologies), ClearRead Xray - Confirm (Riverain Technologies), AI-Rad Companion Brain MR (Siemens Healthineers), AI-Rad Companion Prostate MR (Siemens Healthineers), AI-Rad Companion Chest X-ray (Siemens Healthineers), LungQ (Thirona), LungPrint® Discovery (VIDA), Viz LVO (Viz.ai), Mammography (Zebra Medical Vision), Triage Pneumothorax (Zebra Medical Vision), Bone Health (Zebra Medical Vision), Triage Intracranial Hemorrhage (Zebra Medical Vision), Triage Pleural Effusion (Zebra Medical Vision)
